# (*E*)-2-[(Furan-2-yl)methyl­idene]-7-methyl-2,3,4,9-tetra­hydro-1*H*-carbazol-1-one

**DOI:** 10.1107/S1600536812051203

**Published:** 2013-01-04

**Authors:** A. Thiruvalluvar, R. Archana, E. Yamuna, K. J. Rajendra Prasad, R. J. Butcher, Sushil K. Gupta, Sema Öztürk Yildirim

**Affiliations:** a Postgraduate Research Department of Physics, Rajah Serfoji Government College (Autonomous), Thanjavur 613 005, Tamilnadu, India; b Department of Chemistry, Bharathiar University, Coimbatore 641 046, Tamilnadu, India; c Department of Chemistry, Howard University, 525 College Street NW, Washington, DC 20059, USA; d School of Studies in Chemistry, Jiwaji University, Gwalior 474 011, MP, India; e Faculty of Sciences, Department of Physics, Erciyes University, 38039 Kayseri, Turkey

## Abstract

In the title mol­ecule, C_18_H_15_NO_2_, the atoms in the carbazole unit deviate from planarity [maximum deviation from mean plane = 0.1317 (12) Å]. The pyrrole ring makes dihedral angles of 1.01 (8) and 18.56 (10)° with the benzene and furan rings, respectively. The cyclo­hexene ring adopts a half-chair conformation. In the crystal, pairs of N—H⋯O hydrogen bonds form an *R*_2_^2^(10) ring. Mol­ecules are further linked by C—H⋯O and C—H⋯π inter­actions, forming a three-dimensional network.

## Related literature

For a related structure and the synthesis and applications of carbazole derivatives, see: Archana *et al.* (2010 ▶). For ring conformations, see: Cremer & Pople (1975 ▶). For hydrogen-bond motifs, see: Bernstein *et al.* (1995 ▶).
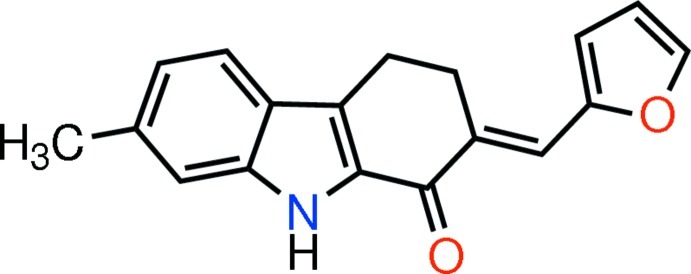


## Experimental

### 

#### Crystal data

C_18_H_15_NO_2_
*M* *_r_* = 277.31
Triclinic, 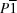

*a* = 6.3925 (3) Å
*b* = 7.9880 (4) Å
*c* = 13.8629 (8) Å
α = 83.151 (5)°
β = 81.649 (4)°
γ = 78.921 (4)°
*V* = 684.28 (6) Å^3^
*Z* = 2
Cu *K*α radiation
μ = 0.70 mm^−1^
*T* = 123 K
0.34 × 0.26 × 0.12 mm

#### Data collection

Agilent Xcalibur Ruby Gemini diffractometer
Absorption correction: multi-scan (*CrysAlis PRO*; Agilent, 2012 ▶) *T*_min_ = 0.816, *T*_max_ = 1.000
4371 measured reflections
2724 independent reflections
2382 reflections with *I* > 2σ(*I*)
*R*_int_ = 0.021

#### Refinement

*R*[*F* ^2^ > 2σ(*F* ^2^)] = 0.047
*wR*(*F* ^2^) = 0.132
*S* = 1.05
2724 reflections
199 parameters
H atoms treated by a mixture of independent and constrained refinement
Δρ_max_ = 0.41 e Å^−3^
Δρ_min_ = −0.29 e Å^−3^

### 

Data collection: *CrysAlis PRO* (Agilent, 2012 ▶); cell refinement: *CrysAlis PRO*; data reduction: *CrysAlis PRO*; program(s) used to solve structure: *SHELXS86* (Sheldrick, 2008 ▶); program(s) used to refine structure: *SHELXL97* (Sheldrick, 2008 ▶); molecular graphics: *ORTEP-3 for Windows* (Farrugia, 2012 ▶) and *PLATON* (Spek, 2009 ▶); software used to prepare material for publication: *SHELXL97* and *PLATON*.

## Supplementary Material

Crystal structure: contains datablock(s) global, I. DOI: 10.1107/S1600536812051203/tk5183sup1.cif

Structure factors: contains datablock(s) I. DOI: 10.1107/S1600536812051203/tk5183Isup2.hkl

Supplementary material file. DOI: 10.1107/S1600536812051203/tk5183Isup3.cdx

Supplementary material file. DOI: 10.1107/S1600536812051203/tk5183Isup4.cml

Additional supplementary materials:  crystallographic information; 3D view; checkCIF report

## Figures and Tables

**Table 1:** Hydrogen-bond geometry (Å, °) *Cg*2 and *Cg*1 are the centroids of the pyrrole (N9/C9*A*/C4*A*/C4*B*/C8*A*) and furan (O11/C12–C15)rings, respectively.

*D*—H⋯*A*	*D*—H	H⋯*A*	*D*⋯*A*	*D*—H⋯*A*
N9—H9⋯O1^i^	0.867 (18)	1.961 (18)	2.8069 (17)	164.9 (17)
C14—H14⋯O1^ii^	0.95	2.55	3.250 (2)	130
C4—H4*B*⋯*Cg*2^iii^	0.99	2.60	3.5176 (16)	154
C17—H17*B*⋯*Cg*1^iii^	0.98	2.89	3.807 (2)	156

Symmetry codes: (i) 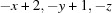; (ii) 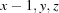; (iii) 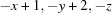.

**Fig. 1.**
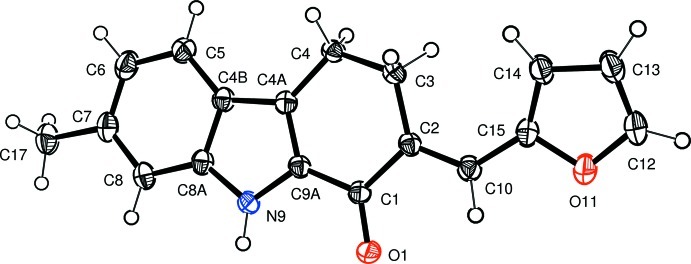
The molecular structure of the title compound, with displacement ellipsoids drawn at the 40% probability level. H atoms are shown as small spheres of arbitrary radius.

**Fig. 2.**
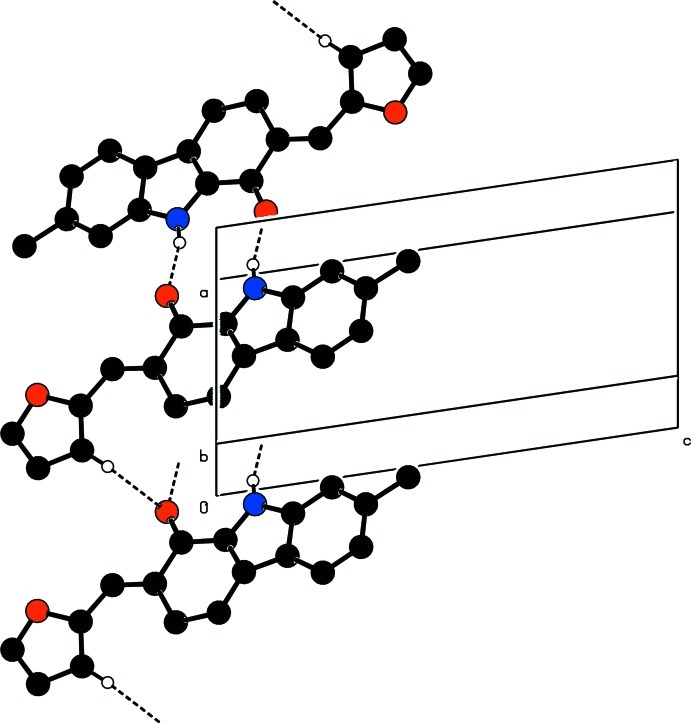
The partial packing of the title compound, viewed approximately down the b axis. Dashed lines indicate hydrogen bonds. H atoms not involved in hydrogen bonding have been omitted.

**Fig. 3.**
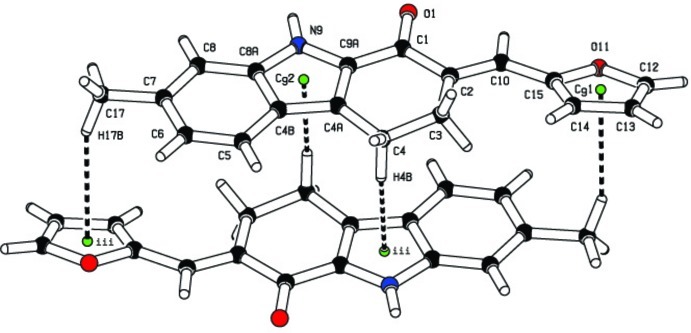
Part of the crystal structure of compound, showing the formation of C—H···π interactions. Symmetry code iii: 1 - x, 2 - y, - z

## supplementary crystallographic information

### Comment

As part of our research (Archana *et al.*, 2010), we have synthesized the title compound (I), and report its crystal structure here.

In the title molecule (Fig. 1), C_18_H_15_NO_2_, the carbazole unit is not planar. Maximum deviation from carbazole mean plane = -0.1317 (12) Å for atom C4. All bond lengths and angles in (I) are normal and comparable with those observed in the related (*E*)-2-(furan-2-ylmethylidene)-8-methyl-2,3,4,9-tetrahydro-1*H*- carbazol-1-one (Archana *et al.*, 2010). The pyrrole ring makes dihedral angles of 1.01 (8) and 18.56 (10)° with the benzene and the furan rings, respectively. The cyclohexene ring adopts a half-chair conformation. The puckering parameters (Cremer & Pople, 1975) are q2 = 0.1372 (15) Å, q3 = 0.1060 (15) Å, Q = 0.1734 (15) Å, θ = 52.3 (5)° and φ = 143.0 (6)°. Intermolecular N9—H9···O1 hydrogen bonds form a *R*^2^_2_(10) (Bernstein *et al.*, 1995) ring motif in the crystal structure (Table 1, Fig. 2). Further, molecules are linked by intermolecular C14—H14···O1, C4—H4B···π, involving the pyrrole (N9/C9A/C4A/C4B/C8A) ring, and C17—H17B···π, involving the furan (O11/C12—C15) ring, interaction to form a three-dimensional architecture (Table 1, Figs 2 & 3).

### Experimental

An equimolar mixture of 7-methyl-2,3,4,9-tetrahydro-1*H*-carbazol-1-one (0.995 g, 0.005 mol) and furan-2-carbaldehyde (0.414 g, 0.005 mol) was treated with 25 ml of a 5% ethanolic potassium hydroxide solution and stirred for 6 h at room temperature. The product precipitated as a yellow crystalline mass, was filtered off and washed with 50% ethanol. A further crop of condensation product was obtained on neutralization with acetic acid and dilution with water. The product was recrystallized from methanol to yield 95% (1.315 g) of the title compound. The pure compound was recrystallized from EtOAc and ethanol.

### Refinement

The H atoms bonded to N9 and C10 were located in a difference Fourier map and refined freely; N9—H9 = 0.867 (18) Å and C10—H10 = 0.964 (19) Å. Other H atoms were positioned geometrically and allowed to ride on their parent atoms, with C—H = 0.95–0.99 Å, and with *U*_iso_(H) = 1.2–1.5*U*_eq_(parent atom).

### Crystal data

**Table:**

C_18_H_15_NO_2_	*Z* = 2
*M**_r_* = 277.31	*F*(000) = 292
Triclinic, *P*1	*D*_x_ = 1.346 Mg m^−^^3^
Hall symbol: -P 1	Melting point: 402 K
*a* = 6.3925 (3) Å	Cu *K*α radiation, λ = 1.54184 Å
*b* = 7.9880 (4) Å	Cell parameters from 2052 reflections
*c* = 13.8629 (8) Å	θ = 5.7–75.5°
α = 83.151 (5)°	µ = 0.70 mm^−^^1^
β = 81.649 (4)°	*T* = 123 K
γ = 78.921 (4)°	Prism, colourless
*V* = 684.28 (6) Å^3^	0.34 × 0.26 × 0.12 mm

### Data collection

**Table:**

Agilent Xcalibur Ruby Gemini diffractometer	2724 independent reflections
Radiation source: Enhance (Cu) X-ray Source	2382 reflections with *I* > 2σ(*I*)
Graphite monochromator	*R*_int_ = 0.021
Detector resolution: 10.5081 pixels mm^-1^	θ_max_ = 75.7°, θ_min_ = 5.7°
ω scans	*h* = −7→7
Absorption correction: multi-scan (*CrysAlis PRO*; Agilent, 2012)	*k* = −8→9
*T*_min_ = 0.816, *T*_max_ = 1.000	*l* = −17→17
4371 measured reflections	

### Refinement

**Table:**

Refinement on *F*^2^	Primary atom site location: structure-invariant direct methods
Least-squares matrix: full	Secondary atom site location: difference Fourier map
*R*[*F*^2^ > 2σ(*F*^2^)] = 0.047	Hydrogen site location: inferred from neighbouring sites
*wR*(*F*^2^) = 0.132	H atoms treated by a mixture of independent and constrained refinement
*S* = 1.05	*w* = 1/[σ^2^(*F*_o_^2^) + (0.0769*P*)^2^ + 0.1562*P*] where *P* = (*F*_o_^2^ + 2*F*_c_^2^)/3
2724 reflections	(Δ/σ)_max_ = 0.001
199 parameters	Δρ_max_ = 0.41 e Å^−^^3^
0 restraints	Δρ_min_ = −0.29 e Å^−^^3^

### Special details

**Table:**

Geometry. Bond distances, angles *etc*. have been calculated using the rounded fractional coordinates. All su's are estimated from the variances of the (full) variance-covariance matrix. The cell e.s.d.'s are taken into account in the estimation of distances, angles and torsion angles
Refinement. Refinement of *F*^2^ against ALL reflections. The weighted *R*-factor *wR* and goodness of fit *S* are based on *F*^2^, conventional *R*-factors *R* are based on *F*, with *F* set to zero for negative *F*^2^. The threshold expression of *F*^2^ > 2σ(*F*^2^) is used only for calculating *R*-factors(gt) *etc*. and is not relevant to the choice of reflections for refinement. *R*-factors based on *F*^2^ are statistically about twice as large as those based on *F*, and *R*- factors based on ALL data will be even larger.

### Fractional atomic coordinates and isotropic or equivalent isotropic displacement parameters (Å^2^)

**Table:**

	*x*	*y*	*z*	*U*_iso_*/*U*_eq_	
O1	0.82745 (16)	0.54561 (14)	−0.10721 (8)	0.0321 (3)	
O11	0.4389 (2)	0.62235 (18)	−0.38788 (8)	0.0462 (4)	
N9	0.77421 (19)	0.66480 (16)	0.08384 (9)	0.0272 (3)	
C1	0.6548 (2)	0.63419 (18)	−0.07581 (10)	0.0256 (4)	
C2	0.4695 (2)	0.68389 (18)	−0.13316 (10)	0.0259 (4)	
C3	0.2549 (2)	0.77564 (19)	−0.08760 (11)	0.0296 (4)	
C4	0.2491 (2)	0.86429 (18)	0.00542 (11)	0.0275 (4)	
C4A	0.4362 (2)	0.79645 (18)	0.05978 (10)	0.0258 (4)	
C4B	0.4742 (2)	0.82727 (18)	0.15417 (10)	0.0279 (4)	
C5	0.3503 (3)	0.9176 (2)	0.23067 (12)	0.0346 (5)	
C6	0.4421 (3)	0.9234 (2)	0.31372 (12)	0.0404 (5)	
C7	0.6555 (3)	0.8434 (2)	0.32410 (12)	0.0375 (5)	
C8	0.7788 (3)	0.7521 (2)	0.25079 (11)	0.0328 (5)	
C8A	0.6863 (2)	0.74409 (18)	0.16619 (10)	0.0285 (4)	
C9A	0.6217 (2)	0.69588 (17)	0.01966 (10)	0.0252 (4)	
C10	0.5039 (2)	0.63565 (19)	−0.22508 (11)	0.0298 (4)	
C12	0.2715 (3)	0.6422 (3)	−0.44181 (13)	0.0492 (6)	
C13	0.0838 (3)	0.6875 (2)	−0.38630 (13)	0.0433 (5)	
C14	0.1333 (3)	0.6999 (2)	−0.29094 (12)	0.0366 (5)	
C15	0.3511 (3)	0.6590 (2)	−0.29413 (11)	0.0317 (4)	
C17	0.7481 (3)	0.8590 (3)	0.41581 (13)	0.0476 (6)	
H3A	0.19484	0.86329	−0.13780	0.0355*	
H3B	0.15695	0.69133	−0.07227	0.0355*	
H4A	0.11559	0.85019	0.04927	0.0329*	
H4B	0.24433	0.98848	−0.01290	0.0329*	
H5	0.20662	0.97310	0.22495	0.0415*	
H6	0.35921	0.98317	0.36572	0.0485*	
H8	0.92199	0.69650	0.25754	0.0394*	
H9	0.900 (3)	0.600 (2)	0.0795 (13)	0.033 (5)*	
H10	0.644 (3)	0.575 (2)	−0.2485 (14)	0.038 (5)*	
H12	0.28626	0.62615	−0.50943	0.0590*	
H13	−0.05517	0.70746	−0.40667	0.0520*	
H14	0.03333	0.73078	−0.23547	0.0439*	
H17A	0.89806	0.80012	0.41094	0.0714*	
H17B	0.74130	0.98028	0.42362	0.0714*	
H17C	0.66510	0.80661	0.47255	0.0714*	

### Atomic displacement parameters (Å^2^)

**Table:**

	*U*^11^	*U*^22^	*U*^33^	*U*^12^	*U*^13^	*U*^23^
O1	0.0224 (5)	0.0448 (6)	0.0290 (5)	−0.0035 (4)	−0.0033 (4)	−0.0073 (4)
O11	0.0401 (7)	0.0717 (9)	0.0272 (6)	−0.0045 (6)	−0.0073 (5)	−0.0117 (5)
N9	0.0233 (6)	0.0337 (6)	0.0261 (6)	−0.0056 (5)	−0.0062 (5)	−0.0041 (5)
C1	0.0221 (7)	0.0301 (7)	0.0259 (7)	−0.0081 (5)	−0.0029 (5)	−0.0020 (5)
C2	0.0240 (7)	0.0291 (7)	0.0260 (7)	−0.0080 (5)	−0.0046 (5)	−0.0007 (5)
C3	0.0254 (7)	0.0345 (7)	0.0297 (7)	−0.0035 (5)	−0.0080 (5)	−0.0040 (6)
C4	0.0235 (6)	0.0297 (7)	0.0294 (7)	−0.0044 (5)	−0.0042 (5)	−0.0033 (5)
C4A	0.0255 (7)	0.0268 (7)	0.0260 (7)	−0.0080 (5)	−0.0029 (5)	−0.0017 (5)
C4B	0.0297 (7)	0.0289 (7)	0.0265 (7)	−0.0079 (5)	−0.0045 (5)	−0.0028 (5)
C5	0.0368 (8)	0.0348 (8)	0.0312 (8)	−0.0035 (6)	−0.0030 (6)	−0.0056 (6)
C6	0.0522 (10)	0.0408 (9)	0.0282 (8)	−0.0065 (7)	−0.0021 (7)	−0.0092 (6)
C7	0.0505 (10)	0.0384 (8)	0.0271 (8)	−0.0136 (7)	−0.0087 (7)	−0.0033 (6)
C8	0.0373 (8)	0.0356 (8)	0.0281 (8)	−0.0094 (6)	−0.0102 (6)	−0.0014 (6)
C8A	0.0317 (7)	0.0298 (7)	0.0260 (7)	−0.0097 (6)	−0.0044 (5)	−0.0025 (5)
C9A	0.0225 (6)	0.0286 (7)	0.0261 (7)	−0.0076 (5)	−0.0046 (5)	−0.0018 (5)
C10	0.0278 (7)	0.0349 (7)	0.0276 (7)	−0.0079 (6)	−0.0043 (5)	−0.0022 (6)
C12	0.0519 (11)	0.0675 (12)	0.0307 (8)	−0.0055 (9)	−0.0171 (8)	−0.0089 (8)
C13	0.0437 (9)	0.0556 (10)	0.0337 (9)	−0.0087 (8)	−0.0169 (7)	−0.0019 (7)
C14	0.0360 (8)	0.0463 (9)	0.0289 (8)	−0.0081 (7)	−0.0099 (6)	−0.0014 (6)
C15	0.0364 (8)	0.0362 (8)	0.0236 (7)	−0.0088 (6)	−0.0047 (6)	−0.0022 (6)
C17	0.0567 (11)	0.0582 (11)	0.0317 (9)	−0.0111 (9)	−0.0140 (8)	−0.0078 (8)

### Geometric parameters (Å, º)

**Table:**

O1—C1	1.2418 (17)	C8—C8A	1.401 (2)
O11—C12	1.367 (2)	C10—C15	1.437 (2)
O11—C15	1.3804 (19)	C12—C13	1.340 (3)
N9—C8A	1.3673 (19)	C13—C14	1.422 (2)
N9—C9A	1.3820 (18)	C14—C15	1.363 (3)
N9—H9	0.867 (18)	C3—H3A	0.9900
C1—C2	1.4867 (19)	C3—H3B	0.9900
C1—C9A	1.4399 (19)	C4—H4A	0.9900
C2—C3	1.513 (2)	C4—H4B	0.9900
C2—C10	1.351 (2)	C5—H5	0.9500
C3—C4	1.536 (2)	C6—H6	0.9500
C4—C4A	1.4857 (19)	C8—H8	0.9500
C4A—C9A	1.3819 (19)	C10—H10	0.964 (19)
C4A—C4B	1.4227 (19)	C12—H12	0.9500
C4B—C5	1.411 (2)	C13—H13	0.9500
C4B—C8A	1.4144 (19)	C14—H14	0.9500
C5—C6	1.375 (2)	C17—H17A	0.9800
C6—C7	1.411 (3)	C17—H17B	0.9800
C7—C17	1.506 (3)	C17—H17C	0.9800
C7—C8	1.382 (2)		
			
C12—O11—C15	106.77 (14)	C10—C15—C14	136.41 (15)
C8A—N9—C9A	107.83 (12)	O11—C15—C10	114.65 (15)
C9A—N9—H9	129.7 (12)	O11—C15—C14	108.81 (14)
C8A—N9—H9	122.2 (12)	C2—C3—H3A	108.00
O1—C1—C9A	121.59 (12)	C2—C3—H3B	108.00
O1—C1—C2	122.91 (13)	C4—C3—H3A	108.00
C2—C1—C9A	115.50 (12)	C4—C3—H3B	108.00
C3—C2—C10	123.32 (12)	H3A—C3—H3B	107.00
C1—C2—C3	120.81 (12)	C3—C4—H4A	109.00
C1—C2—C10	115.81 (12)	C3—C4—H4B	109.00
C2—C3—C4	118.19 (11)	C4A—C4—H4A	109.00
C3—C4—C4A	113.51 (12)	C4A—C4—H4B	109.00
C4B—C4A—C9A	106.39 (12)	H4A—C4—H4B	108.00
C4—C4A—C4B	130.26 (13)	C4B—C5—H5	121.00
C4—C4A—C9A	123.17 (13)	C6—C5—H5	121.00
C5—C4B—C8A	118.99 (13)	C5—C6—H6	119.00
C4A—C4B—C5	134.20 (14)	C7—C6—H6	119.00
C4A—C4B—C8A	106.80 (12)	C7—C8—H8	121.00
C4B—C5—C6	118.52 (16)	C8A—C8—H8	121.00
C5—C6—C7	122.07 (16)	C2—C10—H10	118.6 (12)
C6—C7—C17	119.33 (15)	C15—C10—H10	113.9 (11)
C6—C7—C8	120.43 (16)	O11—C12—H12	125.00
C8—C7—C17	120.25 (17)	C13—C12—H12	125.00
C7—C8—C8A	117.99 (16)	C12—C13—H13	127.00
N9—C8A—C8	129.24 (13)	C14—C13—H13	127.00
C4B—C8A—C8	121.97 (13)	C13—C14—H14	126.00
N9—C8A—C4B	108.78 (12)	C15—C14—H14	126.00
C1—C9A—C4A	125.39 (12)	C7—C17—H17A	109.00
N9—C9A—C1	124.41 (12)	C7—C17—H17B	109.00
N9—C9A—C4A	110.20 (12)	C7—C17—H17C	109.00
C2—C10—C15	127.43 (13)	H17A—C17—H17B	110.00
O11—C12—C13	110.73 (16)	H17A—C17—H17C	109.00
C12—C13—C14	106.59 (17)	H17B—C17—H17C	109.00
C13—C14—C15	107.09 (15)		
			
C15—O11—C12—C13	−0.7 (2)	C9A—C4A—C4B—C8A	0.82 (16)
C12—O11—C15—C10	176.78 (15)	C4—C4A—C9A—N9	174.68 (13)
C12—O11—C15—C14	0.2 (2)	C4—C4A—C9A—C1	−4.3 (2)
C9A—N9—C8A—C4B	−0.07 (16)	C4B—C4A—C9A—N9	−0.89 (16)
C9A—N9—C8A—C8	−178.77 (15)	C4B—C4A—C9A—C1	−179.83 (13)
C8A—N9—C9A—C1	179.56 (13)	C4A—C4B—C5—C6	−178.90 (16)
C8A—N9—C9A—C4A	0.61 (16)	C8A—C4B—C5—C6	1.0 (2)
O1—C1—C2—C3	173.38 (13)	C4A—C4B—C8A—N9	−0.47 (16)
O1—C1—C2—C10	−4.0 (2)	C4A—C4B—C8A—C8	178.34 (14)
C9A—C1—C2—C3	−6.67 (19)	C5—C4B—C8A—N9	179.64 (13)
C9A—C1—C2—C10	176.00 (13)	C5—C4B—C8A—C8	−1.6 (2)
O1—C1—C9A—N9	0.4 (2)	C4B—C5—C6—C7	0.5 (2)
O1—C1—C9A—C4A	179.22 (14)	C5—C6—C7—C8	−1.5 (3)
C2—C1—C9A—N9	−179.53 (13)	C5—C6—C7—C17	178.27 (17)
C2—C1—C9A—C4A	−0.7 (2)	C6—C7—C8—C8A	0.9 (2)
C1—C2—C3—C4	18.4 (2)	C17—C7—C8—C8A	−178.87 (16)
C10—C2—C3—C4	−164.48 (14)	C7—C8—C8A—N9	179.15 (15)
C1—C2—C10—C15	176.62 (14)	C7—C8—C8A—C4B	0.6 (2)
C3—C2—C10—C15	−0.6 (2)	C2—C10—C15—O11	169.91 (15)
C2—C3—C4—C4A	−21.58 (18)	C2—C10—C15—C14	−14.9 (3)
C3—C4—C4A—C4B	−170.30 (14)	O11—C12—C13—C14	0.8 (2)
C3—C4—C4A—C9A	15.28 (19)	C12—C13—C14—C15	−0.6 (2)
C4—C4A—C4B—C5	5.6 (3)	C13—C14—C15—O11	0.24 (18)
C4—C4A—C4B—C8A	−174.32 (14)	C13—C14—C15—C10	−175.19 (18)
C9A—C4A—C4B—C5	−179.32 (16)		

### Hydrogen-bond geometry (Å, º)

Cg2 and Cg1 are the centroids of the pyrrole (N9/C9A/C4A/C4B/C8A) and furan (O11/C12–C15)rings, respectively.

**Table:**

*D*—H···*A*	*D*—H	H···*A*	*D*···*A*	*D*—H···*A*
N9—H9···O1^i^	0.867 (18)	1.961 (18)	2.8069 (17)	164.9 (17)
C14—H14···O1^ii^	0.95	2.55	3.250 (2)	130
C4—H4*B*···*Cg*2^iii^	0.99	2.60	3.5176 (16)	154
C17—H17*B*···*Cg*1^iii^	0.98	2.89	3.807 (2)	156

Symmetry codes: (i) −*x*+2, −*y*+1, −*z*; (ii) *x*−1, *y*, *z*; (iii) −*x*+1, −*y*+2, −*z*.
